# The Transferability of Health Promotion and Education Approaches Between Non-communicable Diseases and Communicable Diseases—an Analysis of Evidence

**DOI:** 10.3934/publichealth.2014.4.182

**Published:** 2014-10-21

**Authors:** David V. McQueen, Erma Manoncourt, Yuri N. Cartier, Irina Dinca, Ülla-Karin Nurm

**Affiliations:** 1 Consultant, Tucker, GA, USA; 2 Consultant, Paris, France; 3 International Union for Health Promotion and Education, Saint-Denis, France; 4 European Centre for Disease Prevention and Control, Stockholm, Sweden

**Keywords:** chronic disease, health communication, health education, health literacy, health promotion, HIV/AIDS, infectious disease, knowledge synthesis translation exchange, non-communicable disease, socioecological model

## Abstract

**Background:**

There is a seeming lack within the public health fields of both research and practice of information sharing across so-called “silos of work”. Many professionals in the public health fields dealing with infectious diseases (IDs) are unaware of the programs and approaches taken by their colleagues in the non-communicable diseases (NCDs) arena, and vice versa. A particular instance of this is in the understanding and application of health promotion approaches. This is a problem that needs to be addressed with the goal of producing the most efficient and effective health promotion approaches to the prevention and control of diseases in general.

**Objectives:**

This project examined health promotion approaches to the prevention of NCDs that could be used in the prevention of IDs.

**Methods:**

A knowledge synthesis and translation perspective was undertaken. We screened and analyzed a wide range of sources that were considered relevant, with particular emphasis on systematic reviews, published articles and the grey literature.

**Results:**

The analysis revealed a diverse health promotion knowledge base for application to IDs. Comprehensive health promotion models were found to be useful. Findings suggest that there are profound similarities for health promotion approaches in both NCDs and IDs. Conclusions: This study revealed gaps in knowledge synthesis to translation. The need for development of intervention and implementation research is considered.

## 1. Introduction

In many areas of public health there is a tendency to work within silos, sharing knowledge only with others in the same field. Health promotion, however, believes it should be multidisciplinary, drawing knowledge and practice from many academic areas. The sharing of knowledge is at the heart of health promotion work and our operating premise in this paper is that many of the workers in the public health fields dealing with infectious diseases (IDs) are unaware of the many approaches taken by their colleagues in the non-communicable diseases (NCDs) arena, and vice versa. This problem needs to be addressed if we desire the most effective approaches to the prevention and control of diseases in general.

It is important to note at the outset a limitation of our study: The separation of diseases into non-communicable and communicable, or chronic and infectious, is often misleading. This separation was mainly based on disease aetiology. The false disease dichotomy has often led to approaches in dealing with the diseases that may be too restrictive and do not take into account the utility of a broader conceptualization of these diseases, and their determinants.

In the prevention of IDs a common approach focuses on awareness, change in attitude, change in behaviour (KAB) and communication at the individual level. Historically health education, in dealing with NCDs, also took an individual-level approach. Over time the concepts of health promotion have taken a broader view that focuses on community level and/or social-cultural changes. We reviewed classical approaches in the health promotion perspective and then concentrated on studies from the past decade. These studies revealed both the complexity of an ecological approach and its utility. However, we found the literature limited by: 1) the lack of a comparative literature that considers both ID and NCD prevention and control outcomes; 2) the limitations of the European context as a base to consider; and 3) the limitations of unpublished or grey literature relevant for our study. Throughout we have been guided by the work of governmental agencies engaged in carrying out systematic reviews of the literature related to health promotion interventions, such as such as the US Centers for Disease Control and Prevention (CDC)'s Community Guide and the National Institute for Health and Care Excellence (NICE)'s reference guides.

At the outset it should be mentioned that many earlier approaches to NCD prevention relate to present day health promotion efforts. Two prime examples are the Framingham Heart Study in the United States and the North Karelia Project in Finland. These well-documented studies show how the application of health promotion principles such as community mobilization and education across sectors led to improved NCD outcomes (notably cardiovascular disease), in part attributable to an approach that adumbrated the ecological approach in health promotion that is discussed below. What is also seen is that these studies reveal the potential transferability of these health-promoting approaches to any disease. Both of these large and influential studies have involved years of research and application, are characterized by the cooperation and work of hundreds of public health scientists, and have resulted in thousands of publications that have influenced the field of health promotion practice. In the case of the Framingham Heart Study, since the mid-20th century nearly 2000 investigators have published some 2500 research articles in peer-reviewed journals. What is particularly critical is that this long-term effort has yielded many useful results in addition to the well-known clinical risk factors, it has articulated and specified in more detail the social, contextual and behavioural factors involved in the causation of CVDs. The extensive literature is well summarized in reviews written over the last six decades [1]–[8].

The North Karelia Project used a settings-based community approach to address the complexity of causality in cardiovascular disease [9]–[14]. It is important to note that North Karelia, from its initiation, was a community-based intervention effort to reduce the very high CVD rates in Finland. It was started in 1972, but was infused with ideas and perspectives arising from health promotion strategies promulgated by the European Region of WHO and captured in the well-known Ottawa Charter for health promotion [15]. The essential health promotion component was the idea of being community-based with the participation of the community, as opposed to the community just being the subject of research. In addition the study focused on interventions with multiple approaches, including marketing, use of primary care physicians, as well as environmental approaches. A widely accepted view is that the North Karelia project demonstrated both the utility and effectiveness of community-based projects with a non-clinical health promoting orientation.

## 2. Materials and Method

Given our thesis that health promotion based approaches carried out to prevent and control non-communicable diseases are equally relevant for application to the prevention and control of communicable diseases, we sought to assess this through an in depth analysis of the extant literature. Initially a broad search of the academic literature was undertaken, considering peer-reviewed studies published mainly in English, Spanish, and French, between January 1990 and July 2013. In particular considerable attention was given to the global literature on health promotion effectiveness [16] and to the broad institutional organizational efforts to evaluate this literature [17]–[20]. An added feature of our analysis was to consider the theoretical health promotion literature that is relevant to the thesis. Also included are health promotion interventions that are not carried out by high-level medical professionals but those that are carried out in non-medical settings and the community. The grey literature was examined to the extent possible.

The potential relevant literature that could be examined is very large. In order to make the project manageable we established some simple guidelines for useful inclusion and exclusion criteria based, in part, on a review of the classical approaches for systematic reviews as developed by PRISMA (Preferred Reporting Items for Systematic Reviews and Meta-Analyses, http://www.prisma-statement.org/index.htm), the Cochrane Collaboration and other established systematic and meta-analysis guidelines. Our approach was not to duplicate these guidelines but to develop the minimum elements appropriate for our analytic study. Whereas these more traditional approaches for systematic reviews are generally framed around scientific rigor, scientific design, and outcome, it was felt that we should concentrate on studies that were already found in the systematic literature but that would be adoptable or transferable to the purpose of addressing IDs. This required a consideration of the similarities found between NCDs and IDs, in causes as well as in long and short term outcomes. The emphasis is on health promotion models, studies and findings related to: 1) modifiable behavioural risk factors that are common to NCDs and IDs; 2) modifiable socio-cultural factors that are common to NCDs and IDs; 3) settings approaches that relate to both NCDs and IDs; 4) community approaches that relate to both NCDs and IDs; and 5) health promoting principles as applicable to NCDs and IDs. Although an important dimension of health promotion, this study does not attempt to focus on policy and governance.

Also underlying our approach is the perspective of health promotion evaluation [21]. In essence, the multidisciplinary nature of the health promotion has led to the evolution of many alternative appropriate views of evaluation. As a result there is no single evaluation methodology that is appropriate for understanding our task; rather, there are several well-developed approaches or lenses that are useful. A widespread finding among evaluative efforts to produce evidence for health promotion is that in approaching community-based health promoting interventions there is the recognition that no two interventions are identical and evaluation solutions are found by carefully combining information on interventions that are similar or analogous in order to fully represent an intervention construct, enhance external validity and usefulness and identify common aspects of effective interventions. This presents a challenge to the traditional use of a rigorous methodological approach such as an RCT. If one takes to constricted methodology, then a major drawback is that many studies do not meet the rigorous methodological criteria established. As a result many areas of intervention yield the unfortunate finding of “insufficient evidence”, which to a great extent is an artefact of methodological criteria that do not deal well with complex interventions. There is thus the growing belief that interventions for which evidence is insufficient should be more appropriately evaluated. The question for evaluation becomes one of whether the evidence is poor or the evidence-seeking behaviour and model are inappropriate. This is a critical point in understanding the nature and usefulness of health promotion interventions in disease prevention, whether chronic or infectious.

These efforts were assisted by an advisory committee consisting of experts in both NCDs and IDs (see Acknowledgements), who were a source of feedback on the progress of the study and who offered advice on what relevant literature and areas to consider.

## 3. Results

### 3.1. Published literature

Our research found that health promotion interventions that are relevant and appropriate to both NCDs and IDs can be found chiefly in the areas of: 1) Health literacy; 2) Participatory Action Research (PAR); 3) Community interventions; 4) Approaches to individual behaviour and social change; 5) Ecological models of intervention and 6) Evaluation of effectiveness and evidence.

#### 3.1.1. Present day health promotion and models

Given the many approaches of present day health promotion, we considered the principal models that are used. There is a large literature based on models or theories. The overall approaches are summarized effectively in Glanz et al. 2008 [22] including the theory of reasoned action, the transtheoretical model and stages of change, behavioural environmental models, social network models, stress and coping models, community models of behavioural change, the diffusion of innovations, theories of organizational change, communication theory and media models, community intervention models, ecological models, and social marketing. A second basic text is provided by Green and Kreuter [23] in four editions published over the past two decades, developing the PRECEDE-PROCEED model widely used by health educators.

**Figure 1.**
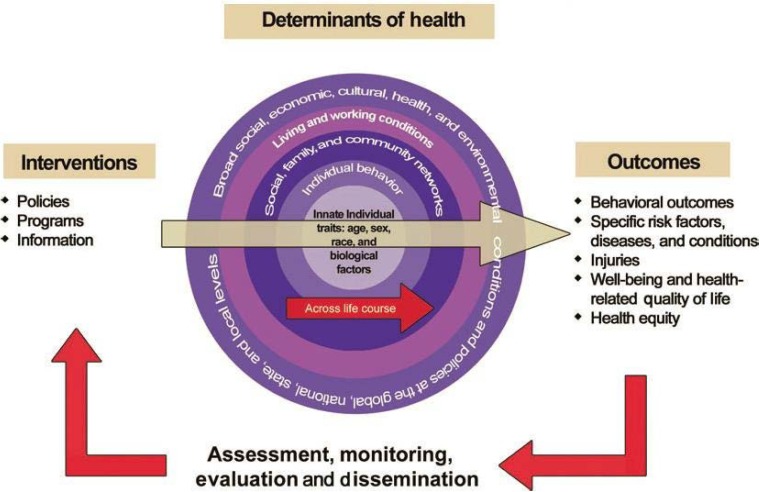
The socioecological model in intervention. Source: National Research Council and Institute of Medicine. (2013). U.S. Health in International Perspective: Shorter Lives, Poorer Health. Panel on Understanding Cross-National Health Differences Among High-Income Countries, Steven H. Woolf and Laud Aron, Eds. Committee on Population, Division of Behavioral and Social Sciences and Education, and Board on Population Health and Public Health Practice, Institute of Medicine. Washington, DC: The National Academies Press.

The origin of the so-called socioecological model is somewhat unclear. By the mid-twentieth century epidemiologists and behavioural scientists were discussing behavioural risk factors, sociocultural risk factors, and environmental factors in complicated disease outcomes [24]. Up to the present such models have become ubiquitous, particularly in the public health literature on diseases with complicated aetiologies, outcomes or high comorbidity. In general all these approaches, whether found in WHO reports and documents or on the US CDC's dedicated web page (CDC 2013), contain many of the same elements. The primary content of these models are reciprocal causal relationships that involve behavioural, social and environmental factors that are highly interrelated and function in a socially structured context. Essentially they are about context, a word that relates highly to present day health promotion theory. Any intervention undertaken to improve health or prevent disease should take a contextual approach. This approach is fundamental to the socio-ecological intervention strategy in public health (Figure 1).

Socioecological models have limitations. They describe the many causal factors operating in relation to any health or disease outcome, but the challenge is to go beyond mere description as a mechanism for interventions. The models are complex and dynamic; therefore, introducing any intervention into this complexity presents many challenges. One would like to be able to manipulate the variables of interest in order to change outcomes, and such complex modelling requires an equally complex understanding of evaluation that stems from many discordant disciplines. Also the use of multilevel interventions has an underlying assumption that the multilevel knowledge base is sound and reasonably developed. Finally, many public health professionals lack the training and skills in the disciplines represented in multilevel models. Thus, while socioecological models have an innate appeal in dealing with the complexities of effecting change in NCD and ID outcomes, their complexity rules them out as a panacea.

#### 3.1.2. Communication approaches

Many health promotion communication-based interventions addressing individual behavioural factors that relate to both IDs and NCDs exist, particularly on smoking, physical activity, diet, and substance abuse. The use of a health promotion lens to summarize this area has been recently extensively reviewed in several chapters in McQueen [24]. In relation to heart diseases and stroke, diabetes, cancer, arthritis and other NCDs, many of which have infectious components, there are multiple and extensive systematic reviews. One illustrative example centred on interventions designed to increase adult fruit and vegetable consumption [26]. Notably, consistent positive effects were seen in studies involving educational counselling, but interventions using telephone contacts or computer-tailored information appeared as a reasonable alternative. Community-based multicomponent interventions also had positive findings.

Similar examples of systematic reviews and a large literature regarding other individual behaviours are extant and examined extensively in the CDC Community Guide and by other review organizations. Although the attention is generally focused on NCDs, most of the approaches would transfer readily to ID prevention. In particular the area of physical activity and ecological approaches to improving physical activity are relevant. Another category within health communication is the area of social marketing, an area with an extensive history in health education. While sharing the established health education efforts concerned with changes in knowledge, attitudes and practices, or KAP, it also links to the broad area of behavioural modification. Those working in social marketing create and use products, programs or interventions to promote health in individuals. There is a strong parallel to the work in consumer research in the public sector. A primary example of this approach in disease prevention, control and health promotion is found in the product of the U.S. CDC entitled “CDCynergy Lite” (http://www.cdc.gov/healthcommunication/cdcynergy/cdcynergylite.htm). The use of such approaches is very useful in the context of highly resourced countries with strong communications infrastructure. While broadly used, its evaluation has been somewhat limited. Effect sizes, i.e. the amount of change in population behaviour, as an example, are often quite small given the costs of funding broad communication programs. While small effect sizes may be quite appropriate for commercial products, it is more problematic for changes related to health behaviours. Nevertheless there are promising results in health communication campaigns with integrated and complex strategies to deliver messages communicated through various channels such as mass media (TV, radio), small media (brochures, posters), and interpersonal communications. Recently attention has begun to focus on the use of social media such as Facebook, Twitter and other internet capabilities. The review and examination of this area are still preliminary. Examined areas by the CDC Community Guide suggest that health communication strategies that include mass media and product distribution interventions are useful when appropriate targeting and segmentation of the population is made. A summation of this evaluation may be found at (http://thecommunityguide.org/healthcommunication/index.html).

#### 3.1.3. Relevance of other disciplines

Health promotion as a field of activity in public health draws on many social science disciplines. It is this broad multidisciplinary background that gives health promotion strategies related to both NCDs and IDs a distinction over more traditional biomedical approaches. Relevant materials in disciplines that relate to health promotion offer other insights into effective interventions. There is a huge extant literature that is relevant. For example, there is a large literature in medical sociology, medical anthropology, medical psychology and health care that utilizes models and explanations that apply to and are used in health promotion approaches. Indeed, many of those who are in the practice of health promotion are trained in these social science disciplines. These multidisciplinary approaches are seen in many large institutional efforts that apply health promotion principles to NCDs and IDs.

A most comprehensive work to consider is that led by Dean Jamison and funded in part by the Bill & Melinda Gates Foundation and is a joint undertaking by the International Bank for Reconstruction and Development / the World Bank, the World Health Organization, and the Fogarty International Center of the National Institutes of Health. The result is a comprehensive document entitled Disease Control Priorities in Developing Countries, 2nd edition [27],[28] that lays out the strategies used in many countries that address a variety of ID-related topics including diarrhoea, vaccination, maternal health, measles, tuberculosis control, and health improvement of the poor using financial incentives. A first finding of importance is that many of these studies show that they can be brought to scale or scaled up—this may be relevant for developed countries as well. A second and perhaps more important and relevant finding is that all of the approaches, though often limited to one disease outcome, ultimately found that the context of the intervention was important and that any approach must begin to take on the multilevel perspective that characterizes the health promotion approach.

In medical sociology, a sub-discipline of sociology has a long history of research, studies and interventions in medical areas and concern with causal factors in disease and illness. A recent and most comprehensive text from this field is the Handbook of the sociology of health, illness and healing [29]. The chapter on “Organizing the Sociological Landscape for the Next Decades of Health and Health Care Research: The Network Episode Model III-R as Cartographic Subfield Guide” [30] is particularly noteworthy. In this chapter a model approach is laid out that is very analogous to many of the socioecological models we have discussed, but it includes a molecular level of genes and proteins. What is notable are the network approach and the inclusion of community systems. What is singularly important about all the details in the Handbook is how much the discussions mirror or are analogous to those in the health promotion field even though they have some differences in background principles and concepts. This field tends to be slightly more concerned with systems, health care, and organizational aspects of health than the health promotion field. Nonetheless it offers valuable insights. Unfortunately the work tends to be more one of understanding and knowledge building than being intervention-oriented.

Finally, a critical background to the dimensions of the health promotion and NCDs in relation to infectious diseases is provided by the notion of disease burden. The “burden” of diseases is seen as a particular challenge for public health across the globe. In the mid-1990s seminal work was carried out and published jointly by the World Health Organization, Harvard University and the World Bank. Of particular note was Volume One in this series of ten entitled The global burden of disease: A comprehensive assessment of mortality and disability from diseases, injuries, and risk factors in 1990 and projected to 2020 [31]. This work lays out in detail the methodology and critical findings of this large study. Recently the Lancet (2012) devoted an entire issue (http://www.thelancet.com/themed/global-burden-of-disease) to the update of this effort which both reinforced the original findings as well as slightly altering the present view of the burden of NCDs. One downside of this huge literature base is that it is descriptive and provides little insight into interventions, although it does show the vast area for intervention potential. Much of this descriptive work continues; for a comprehensive discussion and consideration of the contextual factors from the viewpoints of income inequality, equity and social justice one publication of note is that of Richard Wilkinson and Kate Pickett entitled The Spirit Level: Why More Equal Societies Almost Always Do Better [32]. Also very useful and important is the recent document entitled “U.S. Health in International Perspective: Shorter Lives, Poorer Health” [33] which documents carefully the role of contextual factors in comparison among the OECD countries; it is a powerful illustration that health and illness are not simply a matter of spending money, but that many other factors play a powerful and convincing role.

#### 3.1.4. The importance of HIV–AIDS

One disease area that clearly ties health promotion approaches relevant to NCDs to IDs is HIV–AIDS. This is evidenced through a very large body of literature. It is very relevant because HIV is a communicable disease but at the same time has many characteristics of a chronic disease particularly in terms of longevity, but also because it has many chronic co-morbidities. In addition it is a key disease related to health promotion concepts because of the sociocultural characteristics associated with people living with AIDS and the strong behavioural component of transmission. In addition there are strong parameters related to the social determinants of health and equity. Finally, it is not only health promotion principles that are engaged around this disease; the methodologies of control and management are highly related to health promotion approaches, particularly in the areas of community and health-promoting health care.

The literature relating to young people has been covered very systematically in a review [34] that notes that many behavioural interventions to reduce sexual risk taking behaviours in adolescents show strong effects in the short term, but effects diminish overtime. They also note that this finding may be the result of the failure to take a broader ecological perspective to assess the efficacy of interventions; that is, only interventions that have a multi-causal social perspective will show meaningful results.

One example in particular is noteworthy for our consideration, that of an applied ecological prevention intervention in Brazil. The Brazilian ecological approach is described in detail by Berkman and colleagues [35]. It involves a community grassroots approach that is broad based, engaging the community as well as decision leaders within the community and those that have power with regard to the community (NGOs, trade unions, and government agencies). In the case of the AIDS intervention much effort was made to remove the stigma of AIDS, a common problem in dealing with this disease, by altering some fundamental values that were impeding an effective risk reduction effort. It was an efficacious approach as measured by increased sales and use of condoms. More importantly, incidence rates dropped significantly and even mortality rates began to be affected. As a summation of the vast HIV–AIDS literature on health promotion-type intervention styles it is noteworthy that the ecological approach continues to show strengths and provides an important approach and model for infectious disease prevention and health promotion. Critically, this type of intervention is the one evaluated carefully and recommended by the CDC Community Guide [36]. In addition the CDC produces a Compendium of Evidence-Based HIV Behavioural Interventions [37].

#### 3.1.5. Health literacy

Health literacy is a relatively recent concept in health promotion. Nonetheless, quite an extensive literature has developed. This has recently been reviewed comprehensively [38] with respect to its application in NCD prevention. There is considerable evidence on the effectiveness of health literacy in improving the management of diseases and affecting disease outcomes.

The medical approach to health literacy is largely focused on patient literacy, disease understanding, medication compliance and other biomedical aspects [39],[40]. The health promotion orientation tends to consider health literacy in the context of community development and policy development, although in much of the work undertaken there is a strong emphasis on health education and individual behaviour change [41]. From an educational point of view health literacy tends to follow the integration of health matters into curricula with schools [42],[43]. Further clarification of these distinctions is found in an editorial by Abel [44], and further definitional discussions can be found in many sources [45]–[47].

The relationship between NCDs and health literacy has been explored in depth [48]–[56]. Fortunately there have been multiple systematic reviews of health literacy interventions and although most have been related to NCDs, their findings are clearly applicable to infectious diseases [57]–[62]. The findings of these reviews are quite mixed; however, throughout the reviews are specific applications of high value. Perhaps the most important outcome from the standpoint of application to the communicable disease area is the general agreement that, in principle, policies at national levels are important in addressing the health disparities that exist within geopolitical areas.

The approach can be effective if appropriately applied. However, there is a need for more research to identify the exact efficacy of using different target groups or different settings. It is also clear that there is a need for research on the multidisciplinary aspect of health literacy that integrates it into general research on the idea of “health in all policies”. Finally it is clear that health promotion, and in particular its historical base in health education, is an appropriate field of work to address issues of health literacy.

### 3.2. A selection of grey literature findings

To complement the review of conceptual and theoretical models and communication approaches that could inform health promotion programming for IDs, further exploration was undertaken to identify and learn about the implementation of current health promotion projects—both in Europe and worldwide. The majority of programs studied, based on documentation available, were predominantly oriented towards health promotion activities for NCD prevention and/or generalized health behaviour and social change in marginalized and vulnerable communities. The “Communicate to vaccinate” (COMMVAC) taxonomy [63] provided a final useful framework which was adapted in examining selected health promotion interventions further. In an effort to generate a more complete perspective on lessons learned from the implementation of health promotion programs, the adapted taxonomy facilitated categorization of intervention components into the following implementation categories: a) information or education; b) skills development; c) enabling communication; and d) enhancing community ownership.

Educational activities were a common element across all the health promotion efforts examined; but were particularly characteristic of those interventions that were tailored towards empowering communities/stakeholders to take informed decisions on how to prevent and/or manage a particular chronic disease. These activities were a combination of face-to-face health education sessions, campaigns, as well as print and mediated health messages and materials, including refresher/reminder tools, guides etc., which have been used in various health promotion programs to reach culturally diverse target populations, especially the most marginalized or hardest to reach. In Europe, these groups tend to be ethnic minorities, migrant or immigrant families wherein cultural and social norms differ from the predominant society. Face-to-face interaction with health professionals, community health or family support workers, and/or community resource people/peer educators was the most common intervention implemented. There has also been an effort to implement innovations in health promotion programming such as educational campaigns in informal settings which the population of interest would be likely to frequent, establishing parent cafes (Guardian Angels project), using community health radio for outreach in an urban setting, and developing dramas and exhibitions (Community Health Champions Project) to address a variety of chronic disease prevention issues. These innovative methods of reaching out and attracting the attention of the public and specific vulnerable communities can also be found in ID prevention as exemplified by HIV/AIDS prevention programming. For example, the award-winning “*Soul City It's Real*” television show in South Africa uses drama to tackle discriminatory attitudes and stigma towards infected individuals and bring about a change in individual behaviour and social norms relating to sexual practices while also educating adults and youth about the disease itself [64]. Launched in 1994 and prime-time for the past 17 years, the series is tailored to reflect cultural nuances and social attitudes while promoting change. As a televised multi-media health promotion and social change effort, it now addresses other health-related areas such as domestic violence and led to the establishment of school clubs, youth groups, community-based radio, and educational publications that complement the televised educational process.

In ID prevention, the experiences of tuberculosis programs (Project Hope in Kosovo and the efforts of Norway's Heart & Lung and Diabetes association) have shown the power of peer-to-peer engagement in information dissemination and educational activities as a means of increasing individual and collective behaviour change as well as community acceptance and use of program services. The thrust of the peer-patient interaction goes beyond mere information dissemination to emphasize “understanding” the disease implications, providing the individuals with a sense of self-efficacy (i.e. they could control their lives) and ultimately hope. This experience, replicated by other health promotion programs targeting NCDs, has shown that peer involvement as lay advisors (also known as “experience experts”) has a dual benefit—a) personal empowerment of the peer herself and b) increased receptivity to health promotion and/or disease prevention messages by the intended stakeholder and therefore, increased motivation to change negative behaviour.

Skills development interventions were targeted both to health providers and/or local communities/parents/young people via workshops, courses, or training sessions, and tended to have a two-fold purpose: a) to help them internalize their own knowledge about disease prevention or health promotion and b) to prepare them to help others. These activities were especially prominent in programs that focused on engaging community members and local stakeholders in health promotion and/or disease prevention activities. For example, the Mit MiGranten Für Migranten program engaged fairly well-integrated persons with immigrant backgrounds who received health instruction and then later served as community contact persons and health promotion advocates to different ethnic/cultural groups. In the global polio eradication initiative, with an intense focus on three endemic countries (Nigeria, Pakistan and Afghanistan), this capacity development approach mirrors and has been refined to focus on strengthening both the technical and interpersonal communication skills of polio vaccinators while simultaneously engaging local residents as social mobilisers to increase community outreach and demand for vaccination [65],[66].

Interventions that enabled communication sought to minimize cross-cultural misunderstandings of health promotion content and maximize cultural acceptability of the actions being promoted to prevent disease among culturally diverse communities. Whether working with Roma or immigrant communities, one distinguishing characteristic of the health promotion programs reviewed was their efforts in communicating health promotion advice in a culturally relevant and sensitive manner. In some instances, this involved developing multi-language educational materials with relevant images and cultural contexts, which could be tailored to specific ethnic or language minority communities. Recognizing the importance of communicator characteristics in getting a health message across, some of the health promotion programs engaged local community members as lay advisors (who depending upon the program, were referred to as peer educators/volunteers, community health champions, experience experts, etc.) who helped facilitate knowledge transfer, were actively involved in promoting behaviour and social change initiatives, and provided advice and support to families as needed in the change process. In the United States, the Centre for Disease Control's Racial and Ethnic Approaches to Community Health (REACH) program was specifically designed and implemented with this concept in mind. In its cancer prevention work, Barefoot Health Workers Project (Wales) worked with honorary mediators which led to the enabling of “own language” promotion of key health messages on breast screening.

Enhancing community ownership of and buy-in for health promotion and related behaviour change was an approach used by many of the projects reviewed. Since the majority of the health promotion programs were community-based, they often used “intermediaries,” either community health workers (professionals) or local residents (volunteers as peer educators) to engage in health promotion dialogue and interactions with a hard-to-reach population in need. As a result, the health promotion programs were more effective in penetrating and reaching the most vulnerable and marginalized—bringing visibility to those forgotten and hearing the voices of those who had been left behind from other, wider health promotion initiatives. Using an empowerment principle as its base, the Community Health Champions project has been an ambitious endeavour that involves local community members in determining how best to address health issues of local concern by a) engaging community members who are disease survivors or already living with the disease as part of their health promotion team either formally or informally; b) coupling lay advisors/peers' familiarity with local issues with health promotion and disease prevention objectives; and c) involving community resource people in needs assessment and program design and monitoring. The “low barrier method” was another common factor in health promotion interventions, as exemplified by the Northside Community Health Initiative (NICHE) project in Ireland, which brings complementary health services to the community, rather than vice versa. The project is run by local staff, with local management and addresses a local agenda. This approach has contributed to a snowball effect of wider community acceptance of disease prevention and health promotion actions. The community-based approach has also been integrated effectively in disease prevention efforts against avian influenza outbreaks wherein accelerated educational programmes and communication activities were designed and implemented to reach out to high-risk communities and the general public to prevent their spread.

## 4. Discussion: challenges and needs for implementation research in this area

Although the grey literature review identified four salient common lessons that have design implications for health promotion programs focused on ID prevention, there is a dearth of accessible information about behaviour and social change dimensions of preventing the spread of IDs, health promotion good practices, case studies and lessons learned (i.e. concrete program information) on the professional organizations' websites, with the exception of EuroHealthNet. At times, when a potentially interesting program is described, the link to fuller documentation is no longer active and a Google search could not surface relevant information. A cursory scan of national public health departments or institutes in selected European countries also revealed a paltry amount of information about actual health programs being implemented (theoretical framework guiding program design, implementation strategy etc.), especially health promotion interventions. In cases where health program was mentioned, little or no documentation was provided. Often, these programs indicated that monitoring and evaluation efforts had been undertaken but only internal documents were available.

There is extensive grey literature documentation on health promotion program design, strategies and activities related to physical activity, exercise and smoking cessation, which is in direct contrast to what is available about health promotion linked to IDs or NCDs. This review surfaced a serious problem in identifying and accessing comprehensive descriptions, including conceptual/theoretical frameworks for project design, and/or discussions of relevant on-the-ground activities and programmatic details of health promotion interventions in the aforementioned areas. On public health-related websites, one is more likely to find information on disease prevention than health promotion; but even then, it also tends to be focused on control and management of IDs or NCDs, *per se.* Additionally, if health promotion programs are mentioned on these sites, very little information is provided—in essence, health promotion practice is “invisible.” In comparison, in health promotion-related websites, cursory information is provided (i.e. IUHPE, SOPHE) but more comprehensive details about program descriptions, design and other implementation data are not always available. A similar pattern emerges in reviewing the conference schedule and report/proceedings in health related international conferences (Social Determinants of Health—Rio and High Level Meeting on NCDs, Public Health Association Annual Conferences) where health promotion has minimum mention but focus tends to be health policy, systems development etc.

Based on conference proceedings, there is evidence that there are health promotion programs in Eastern Europe but information about health promotion practice—both for IDs and NCDs—in this region is most difficult to find; and hence it may be under-represented in the review. There are several potential explanations for these lacunae. First, health promotion as a discipline may still be relatively young in this region compared to a stronger more medical approach to health programs; so relatively few health promotion activities documented. For example, during the EuroHealthNet website review of “good practices,” some of the Eastern European case examples tended to be more health systems-focused or institutional capacity-building, rather than a reflection of health promotion interventions. Secondly, language differences may impede translation of potentially available documentation for a wider dissemination through social professional networks such as EuroHealthNet and IUHPE.

In light of the above situations, there is a need to support implementation research for health promotion programming relevant to both IDs and NCDs in a much more proactive and systematic manner than current efforts. This would entail national governments, civil society organizations, and international agencies committing to provide sufficient funds to finance program audits, operations research, and program evaluations of health promotion projects/interventions. As such, there should be identified funds which are earmarked (at international and ideally at national level, too) for such activities and incorporate them in requests for proposals and/or provide seed money opportunities to health promotion programs to complete this type of documentation. A second step would also be to support the creation of an “e-repository” of systematic documentation or virtual library of health promotion practice, including good practices, lessons learnt, etc. in the field of IDs that could be consulted by practitioners, policy-makers and academicians alike.

## 5. Conclusion

In addition to the availability of evidence for effective health promotion that is found in the scientific literature and the multitude of conceptual models and theoretical frameworks that have been developed to inform health promotion work in general, there still remains the need to expand the collective knowledge about the evolving, concrete realities (strengths, weaknesses, opportunities and threats) of health promotion practice, with a particular emphasis on interventions reaching out to cultural/social minorities and marginalized populations. The systematic collection and syntheses of these project experiences and program findings would be an invaluable contribution to the collective learning of what works, the applicability of different theories and frameworks in different socio-cultural contexts, and the replicability of different health promotion strategies across the infectious to non-communicable disease continuum. The identification of “good practices” and “lessons learnt” in health promotion could set the foundation for a European health promotion community of practice (e.g. interest group, informal network) for communicable diseases.
